# Tfeb-Mediated Transcriptional Regulation of Autophagy Induces Autosis during Ischemia/Reperfusion in the Heart

**DOI:** 10.3390/cells11020258

**Published:** 2022-01-13

**Authors:** Jihoon Nah, Eun-Ah Sung, Peiyong Zhai, Daniela Zablocki, Junichi Sadoshima

**Affiliations:** 1 Department of Cell Biology and Molecular Medicine, Rutgers New Jersey Medical School, Newark, NJ 07103, USA; namoogun@naver.com (J.N.); es1006@gsbs.rutgers.edu (E.-A.S.); zhaipe@njms.rutgers.edu (P.Z.); danizablocki@yahoo.com (D.Z.); 2 School of Biological Science, Seoul National University, 1 Gwanak-ro, Gwanak-gu, Seoul 08826, Korea; 3 Cardiovascular Research Institute, Rutgers New Jersey Medical School, 185 South Orange Ave., MSB G609, Newark, NJ 07103, USA

**Keywords:** autophagic cell death, autosis, Tfeb, ischemia/reperfusion

## Abstract

Autosis is a unique form of cell death with characteristic morphological and biochemical features caused by dysregulated autophagy. Autosis is observed in the heart during the late phase of ischemia/reperfusion (I/R), when marked accumulation of autophagosomes is induced. We previously showed that the excessive accumulation of autophagosomes promotes autosis in cardiomyocytes. Although the inhibition of autophagic flux via the upregulation of Rubicon induces the accumulation of autophagosomes during I/R, it appears that additional mechanisms exacerbating autophagosome accumulation are required for the induction of autosis. Here, we show that Tfeb contributes to the induction of autosis during the late phase of I/R in the heart. During myocardial reperfusion, Tfeb is activated and translocated into the nucleus, which in turn upregulates genes involved in autophagy and lysosomal function. The overexpression of Tfeb enhanced cardiomyocyte death induced by a high dose of TAT-Beclin 1, an effect that was inhibited by the downregulation of Atg7. Conversely, the knockdown of Tfeb attenuated high-dose TAT-Beclin1-induced death in cardiomyocytes. Although the downregulation of Tfeb in the heart significantly decreased the number of autophagic vacuoles and inhibited autosis during I/R, the activation of Tfeb activity via 3,4-dimethoxychalcone, an activator of Tfeb, aggravated myocardial injury during I/R. These findings suggest that Tfeb promotes cardiomyocyte autosis during the late phase of reperfusion in the heart.

## 1. Introduction

Autophagy is an evolutionarily conserved self-digestion system. Double membrane vacuoles selectively or non-selectively engulf cytoplasmic materials and fuse with lysosomes to degrade the contained cargo materials. Autophagy is tightly regulated by autophagy-related (Atg) genes and other non-Atg genes [1,2]. Autophagy generally acts adaptively both at baseline and in response to stress by either supplying materials for cellular maintenance or survival or eliminating non-functional or unnecessary proteins and organelles [3,4]. However, dysregulated autophagy could trigger cell death [5]. Despite extensive research into the mechanisms that cause autophagic cell death, the detailed molecular mechanisms involved remain elusive.

Autosis is an autophagy-dependent non-apoptotic form of cell death that has unique morphological and biochemical features. Unlike in typical autophagy, cells with autosis exhibit electron-dense mitochondria at an early stage and ballooning of the perinuclear space at a late stage, as indicated by electron microscopic analyses. Autosis is facilitated not only through the stimulation of the autophagy core machinery but also by Na^+^, K^+^-ATPase, a non-autophagy protein [6,7]. Na^+^, K^+^-ATPase is a membrane protein that is located on the plasma membrane, perinuclear membrane, mitochondria and endosomes and regulates cellular Na^+^/K^+^ gradients and calcium signaling [8]. The genetic or pharmacological inhibition of Na^+^, K^+^-ATPase was shown to decrease autosis in a rat model of cerebral hypoxia–ischemia injury and ischemia/reperfusion (I/R) in the mouse heart [6,9]. A recent study showed that Na^+^, K^+^-ATPase physically interacts with Beclin 1, and that this interaction is induced by autosis-inducing conditions, including starvation, ischemia, exercise and I/R [7]. However, how this interaction regulates autosis remains to be elucidated.

Recently, we reported that autosis is triggered during the late stage of I/R in the heart and contributes to infarction injury. In that study, we showed that the upregulation of Rubicon during the late phase of reperfusion contributes to autosis in the mouse heart [9]. Rubicon is a Beclin 1-interacting protein that inhibits autophagic flux, especially in the autophagosome–lysosome fusion process [10]. Autophagic flux is activated during ischemia and up to 6 h of reperfusion. However, it is inhibited thereafter due to the upregulation of Rubicon. Since autophagosomes continue to be made even after autophagic flux is inhibited, autophagic vacuoles accumulate drastically thereafter, leading to a shortage of intracellular membranes, the cellular sources of autophagosomes [9,11]. Although Rubicon contributes to the drastic accumulation of autophagosomes by inhibiting autophagic flux, the upregulation of Rubicon alone does not induce autosis. Thus, the molecular mechanisms that induce the drastic accumulation of autophagosomes during the late stage of reperfusion remain to be clarified.

Transcription factor EB (Tfeb) is a master regulator for lysosomal biogenesis and autophagy [12]. Tfeb belongs to the larger family of basic helix–loop–helix leucine zipper transcription factors. Tfeb binds to a coordinated lysosomal expression and regulation (CLEAR) motif (GTCACGTGAC) [13] and regulates the expression of lysosomal genes [14]. Tfeb also regulates the expression of autophagy-regulating genes, including autophagy initiators (Beclin 1, Atg9B and Wipi1), elongators (MAP1LC3B, ATG5 and GABARAP) and lysosome fusion mediators (UVRAG and RAB7) [13]. Thus, the activation of Tfeb triggers the upregulation of autophagy and lysosomal genes and eventually activates autophagy. Tfeb is activated during I/R in kidneys and in macrophages in the heart [15,16]. However, the role of Tfeb in regulating cell death and organ injury during myocardial reperfusion remains unknown.

Our preliminary investigation suggested that Tfeb is upregulated during the late phase of reperfusion, when Rubicon is upregulated and autosis is observed, in the mouse model of I/R. We reasoned that the simultaneous upregulation of the factor promoting autophagy and that inhibiting autophagic flux would induce a condition where a marked accumulation of autophagosomes may take place. Thus, the aim of this study was to show that Tfeb is involved in the stimulation of cardiomyocyte autosis during the late phase of reperfusion.

## 2. Materials and Methods

### 2.1. Ischemia/Reperfusion In Vivo

C57BL/6J mice were obtained from the Jackson Laboratory. Mice were housed in a temperature-controlled environment with 12 h light/dark cycles, where they received food and water ad libitum. Three-month-old C57BL/6J mice were anesthetized via an intraperitoneal injection of pentobarbital sodium (60 mg/kg). A rodent ventilator (Minivent; Harvard Apparatus Inc., Holliston, MA, USA) was used with 65% oxygen during the surgical procedure. The animals were kept warm using heat lamps. Rectal temperature was monitored and maintained between 36.8 and 37.2 °C. The chest was opened using a horizontal incision at the fourth intercostal space. Ischemia was achieved by ligating the anterior descending branch of the left coronary artery (LAD) using an 8-0 prolene suture, with silicon tubing (1 mm OD) placed on top of the LAD, 2 mm below the border between the left atrium and left ventricle (LV). Ischemia was confirmed using ECG change (ST elevation) and color change of the myocardium. After occlusion for 30 min, the silicon tubing was removed to achieve reperfusion. When recovered from anesthesia, the mice were returned to their cages and housed in a climate-controlled environment.

### 2.2. Measurement of Infarct Size

After I/R, the animals were anesthetized and intubated, and the chest was opened. To demarcate the ischemic area at risk (AAR), Alcian blue dye (1%) was perfused into the aorta and coronary arteries. The hearts were excised, and LVs were sliced into 1 mm thick cross-sections. The heart sections were then incubated with a 1% triphenyl tetrazolium chloride (TTC) solution at 37 °C for 15 min. The infarct area (white), the AAR (not blue) and the total LV area from both sides of each section were measured using ImageJ software, and the values obtained were averaged. The percentage of the area of infarction and AAR of each section was multiplied by the weight of the section and then totaled from all sections. AAR/total LV and infarct area/AAR were expressed as percentages [17].

### 2.3. Cardiomyocyte Cultures

Primary cultures of neonatal rat cardiomyocytes were prepared from 1-day-old Charles River Laboratories (Crl)/Wistar Institute (WI) BR-Wistar rats (Harlan Laboratories, Somerville, NJ, USA) as described previously [18]. A cardiomyocyte-rich fraction was obtained via centrifugation through a discontinuous Percoll gradient.

### 2.4. Viability of the Cells

The viability of the cells was measured using CellTiter Blue assays (Promega, Madison, WI, USA). In brief, cardiomyocytes were seeded onto 96-well dishes. The cells were preincubated with the indicated adenovirus for 48 h. Viable cell numbers were measured after Tat-Scrambled or Tat-Beclin 1 treatment. The CellTiter Blue assays were performed according to the supplier’s protocol. Staining of cultured cardiomyocytes with SYTOX green nucleic acid stain (Invitrogen, Thermo Fisher Scientific, Waltham, MA, USA) to evaluate cell death was conducted according to the manufacturer’s instructions.

### 2.5. Tat-Beclin 1

The Tat-Beclin 1 amino acid sequence was YGRKKRRQRRGGVWNATFHIWHD. The control peptide, Tat-Scrambled (TS), consisted of the Tat protein transduction domain, a GG linker, and a scrambled version of the C-terminal 11 amino acids of Tat-Beclin 1 (YGRKKRRQRRGGWNHADHTFVWI). For the induction of autophagy in vitro, cardiomyocytes were washed with PBS (–) and treated with peptides dissolved in OPTI-MEM acidified with (0.15% *v*/*v*) 6 N HCl [19].

### 2.6. Electron Microscopy

Conventional electron microscopy (EM) was performed as described previously [17]. The average number of vacuoles or electron-dense mitochondria was calculated per 400 µm^2^ area.

### 2.7. Real-Time Quantitative Polymerase Chain Reaction (qPCR)

Total RNA was extracted from mouse hearts using the RNeasy Plus Universal Kit (QIAGEN). Reverse transcription was performed from 1 ug of RNA PrimeScript RT Master Mix (Takara). The list of primers for qPCR are presented in Table 1.

### 2.8. Immunoblot Analyses

The methods used for the preparation of cell lysates from in vitro and in vivo samples and for immunoblot analyses have been previously described [20]. The antibodies used included TFE3 (HPA023881, MilliporeSigma, Burlington, MA, USA), ATP6V1A (17115-1-AP, Proteintech, Rosemont, IL, USA), ATP6V1B1 (A-6427, Invitrogen, Waltham, MA, USA), Histone H3 (4499, Cell Signaling Technology, Danvers, MA, USA), LaminB1 (13435, Cell Signaling Technology), pTfeb (Ser142) (AF3845, Affinity Biosciences, Cincinnati, OH, USA), Atg7 (8558, Cell Signaling Technology), GAPDH (2118S, Cell Signaling Technology), αTubulin (T6199, Sigma-Aldrich, Burlington, MA, USA) and Tfeb (A303-673A, Bethyl Laboratories, Montgomery, TX, USA; sc-48784, Santa Cruz Biotechnology, Dallas, TX, USA).

### 2.9. Immunostaining Analysis

The heart was isolated, fixed with 4% phosphate-buffered paraformaldehyde (PFA), embedded in paraffin and cut into 10 μm thick sections. Deparaffinized sections of the heart were stained with anti-Tfeb antibody and DAPI. Samples were observed in a blinded manner using Nikon A1RSI confocal microscopy. The percentage of Tfeb-positive nuclei (%) was calculated as the number of Tfeb (+) nuclei/total number of DAPI (+) nuclei.

### 2.10. Statistical Analysis

Statistical analyses were conducted using the GraphPad Prism 8.0 software program. Data are expressed as the mean ± standard deviation of the indicated number of experiments or the mean ± standard error of the mean for the indicated number of mice. The difference in means between 2 groups or multiple groups was evaluated using the unpaired Student’s t test or one-way ANOVA, respectively. Post-test comparisons for multiple analyses were performed using the Dunnett’s test. *p* values were 2-sided, and *p* values of <0.05 were considered statistically significant.

## 3. Results

### 3.1. The Tfeb-Mediated Autophagy–Lysosome Pathway Is Persistently Upregulated during I/R

**Figure 1:**
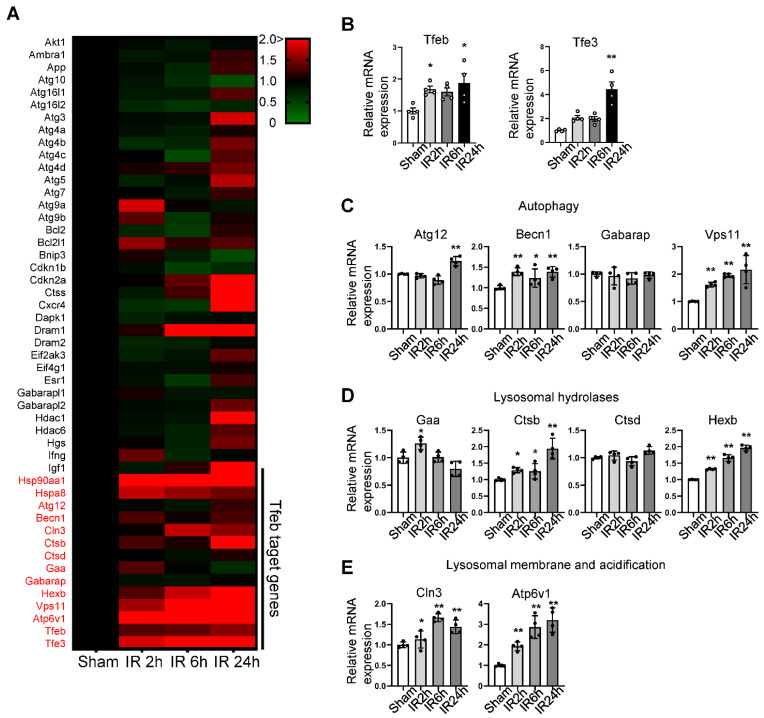
Pathway-focused qPCR array analysis shows a comparison of the autophagy and lysosomal gene profile in sham and I/R heart samples. An autophagy-related, pathway-focused qPCR array was performed using primers for 75 autophagy-related genes. Three-month-old C57BL/6J mice were subjected to 30 min of ischemia followed by reperfusion for the indicated times, and whole heart lysates were analyzed via qPCR analysis. (**A**) Heat map showing changes in gene expression levels relative to sham samples, indicated by color change. (**B**–**E**) Graphs showing relative gene expression levels of Tfeb and Tfe3, (**B**) autophagy-related (Atg) genes, (**C**) lysosomal hydrolase genes (**D**) and lysosomal-membrane- and acidification-related genes (**E**). Mean ± S.E., n = 4; * *p* < 0.05, ** *p* < 0.01 versus sham; 1-way ANOVA with Dunnett’s post hoc test.

Autosis is activated during the late phase of reperfusion, when the number of autophagic vacuoles is drastically increased [9]. Although the inhibition of autophagic flux caused by the upregulation of Rubicon facilitates autophagosome accumulation, additional mechanisms facilitating the dysregulated production of autophagosomes may exist. We therefore conducted PCR array experiments to evaluate how autophagy-related genes are affected during the late phase of reperfusion in the mouse heart (Table 1). Interestingly, Tfeb family transcription factors, including Tfeb and Tfe3, and many Tfeb-regulated genes are upregulated from the early to late stages of I/R (Figure 1A). Confirming the results of the PCR array analyses, qPCR analyses showed that known Tfeb target genes involved in autophagy and lysosome function were upregulated during I/R (Figure 1B–E).

**Figure 2:**
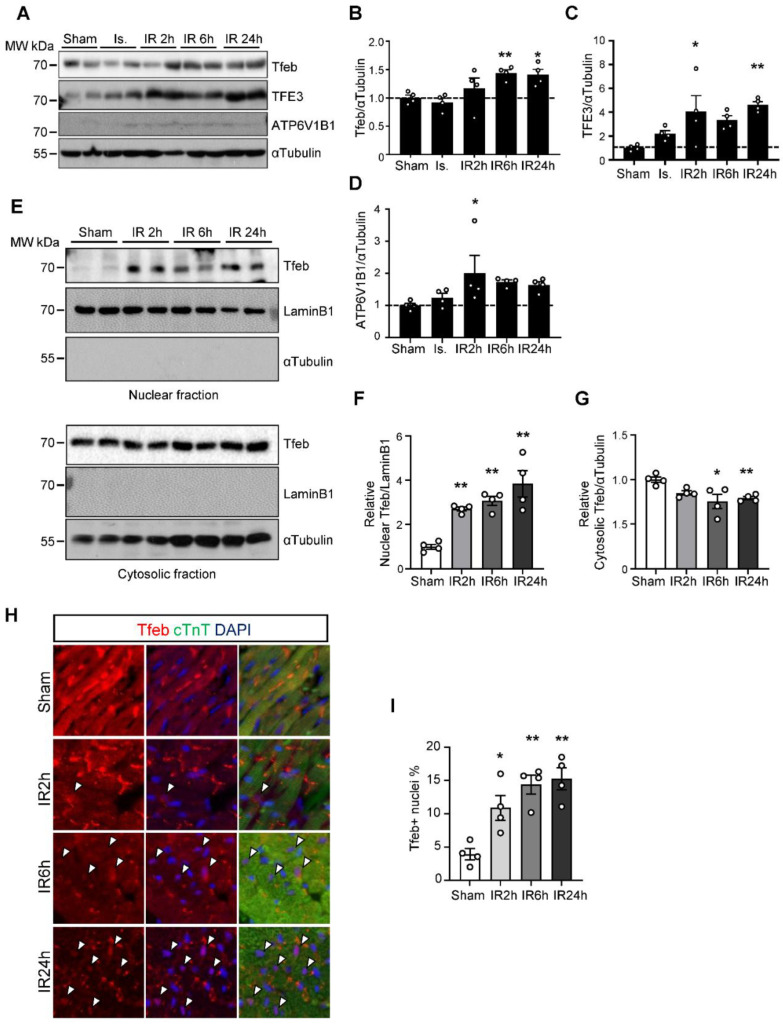
Tfeb is translocated into the nucleus in response to I/R. Three-month-old mice were subjected to 30 min of ischemia with reperfusion for the times indicated. (**A**–**D**) Heart lysates were analyzed via Western blot using anti-Tfeb, anti-TFE3, anti-ATP6V1B1 and anti-αTubulin antibodies. (**A**) The ratios of Tfeb to αTubulin (**B**) TFE3 to αTubulin (**C**) and ATP6V1B1 to αTubulin (**D**) were quantified (mean values ± S.E., n = 4; * *p* < 0.05, ** *p* < 0.01 versus sham). (**E**–**G**) The mouse heart samples were subjected to nuclear fractionation. Both the nuclear and cytosolic fractions were analyzed via Western blot analyses using anti-Tfeb, anti-LaminB1 and anti-αTubulin antibodies (**E**). The relative ratios of nuclear Tfeb (**F**) and cytosolic Tfeb (**G**) were quantified (mean values ± S.E., n = 4; ** *p* < 0.01 versus sham). (**H**,**I**) The heart samples were subjected to immunostaining using anti-Tfeb antibody. Representative immunofluorescent DAPI (blue) and Tfeb (green) staining of sections from the infarction area in the mouse hearts (**H**). Nuclear Tfeb was determined by calculating the percentage of Tfeb and DAPI double-positive cells (measured in 3 different view fields per section, 3 sections per mouse with 4 mice per group, mean values ± S.E., * *p* < 0.05 ** *p* < 0.01 versus sham) (**I**).

Next, we evaluated whether Tfeb is activated during I/R in the mouse heart. Consistent with the result of PCR array analyses, qPCR analyses showed that the expression level of Tfeb and Tfe3 were both increased in response to I/R (Figure 1B). In addition, both Tfeb and TFE3 were upregulated at the protein level as early as 2–6 h after reperfusion and remained increased at 24 h (Figure 2A–C). ATP6V1B1 also tended to be upregulated at the protein level during the late phase of reperfusion (Figure 2D). Tfeb is translocated from the cytosol/lysosome to the nucleus in response to stress [21]. Subcellular fractionation and immunoblot analyses revealed that Tfeb is increased in the nuclear fraction but decreased in the cytosolic fraction, consistent with nuclear translocation, in response to reperfusion compared to after a sham operation and that it remains increased in the nuclear fraction during the late reperfusion phase (Figure 2E–G). Immunostaining of heart sections also showed that the expression of Tfeb in the nucleus, as indicated by the ratio of Tfeb-positive nuclei/total nuclei, is increased significantly during reperfusion in the heart (Figure 2H,I). Taken together, these results suggest that Tfeb is activated in response to reperfusion and remains activated during the late reperfusion phase.

### 3.2. Tfeb Facilitates Tat-Beclin 1-Induced Autosis in Neonatal Rat Cardiomyocytes (NRCMs)

**Figure 3:**
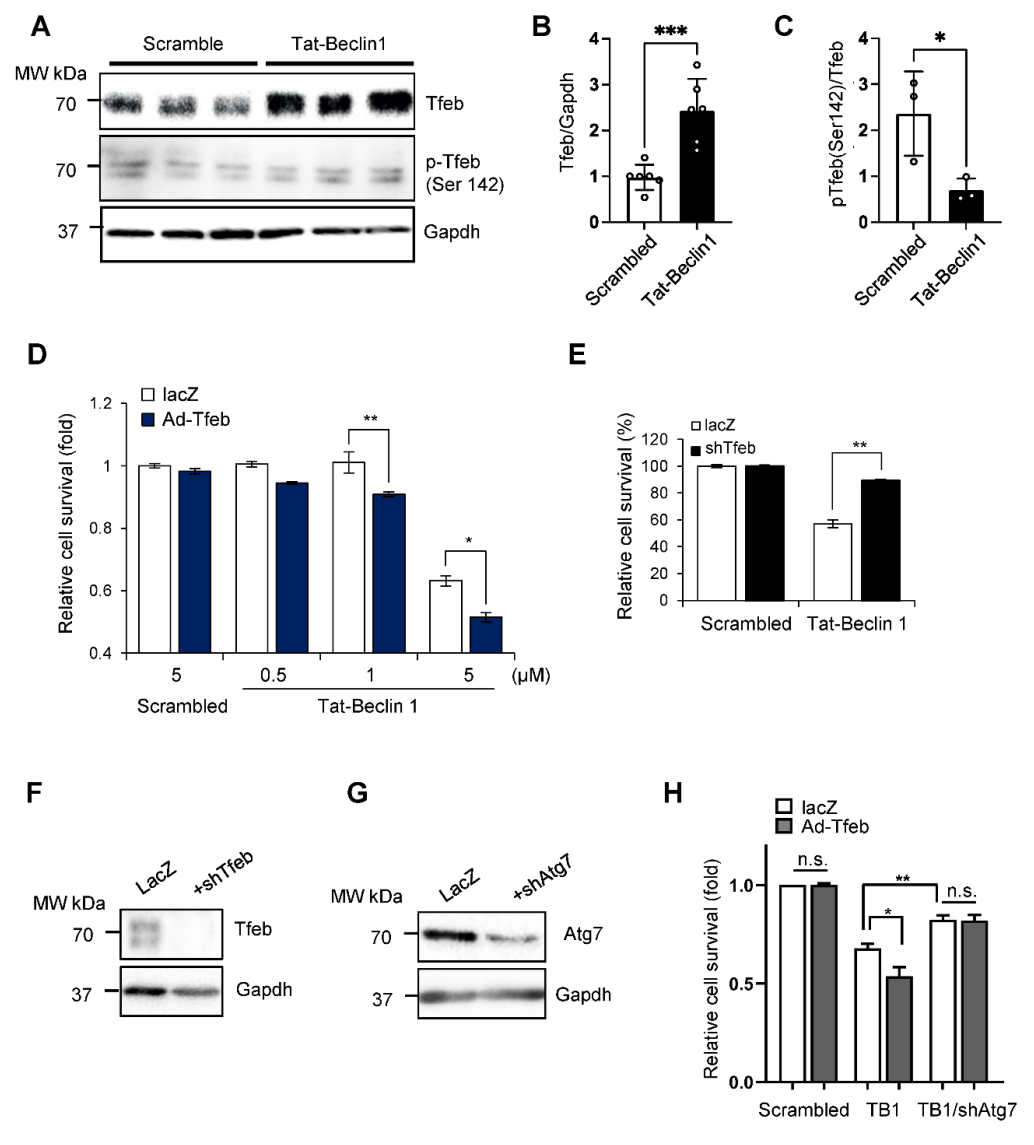
Tat-Beclin1-induced autosis is regulated by Tfeb modulation in NRCMs. (**A**–**C**) NRCMs were treated with 5 µM Scrambled or Tat-Beclin 1 for 6 h and analyzed via Western blotting using anti-Tfeb, anti-pTfeb (Ser142) and anti-Gapdh antibodies (**A**) The relative ratios of Tfeb to Gapdh (**B**) and pTfeb to Tfeb (**C**) were quantified (mean values ± S.E., n = 6 for Tfeb, n = 3 for p-Tfeb; * *p* < 0.05, *** *p* < 0.001). (**D**,**E**) NRCMs were transduced with either Ad-lacZ or Ad-Tfeb for 24 h (**D**) or Ad-lacZ or Ad-shTfeb for 48 h (**E**) and then treated with 5 µM Scrambled or 0.5, 1 or 5 µM Tat-Beclin 1 for 3 h. Cell death was quantified via CellTiter-Blue assays (mean values ± S.D., n = 16; * *p* < 0.05, ** *p* < 0.01, not significant (n.s.)) (**D**,**E**). (**F**,**G**) Knockdown of Tfeb by Ad-shTfeb was confirmed via immunoblot analyses, using anti-Tfeb and anti-Gapdh antibodies (**F**). Knockdown of Atg7 by Ad-shAtg7 was confirmed via immunoblot analyses, using anti-Atg7 and anti-Gapdh antibodies (**G**). (**H**) NRCMs were transduced with either Ad-lacZ or Ad-Tfeb with or without Ad-shAtg7 as indicated for 48 h and then treated with 5 µM Scrambled or Tat-Beclin 1 for 3 h. Cell death was quantified via CellTiter-Blue assays (mean values ± S.D., n = 16; * *p* < 0.05, ** *p* < 0.01, not significant (n.s.)).

We next evaluated how the upregulation of Tfeb affects the survival and death of cardiomyocytes when autophagy is pre-stimulated by Tat-Beclin1, mimicking the conditions during I/R. Interestingly, Tat-Beclin 1 treatment significantly increased the level of Tfeb in NRCMs (Figure 3A,B). Moreover, the ratio of Tfeb phosphorylated at Ser142 to total Tfeb was decreased by Tat-Beclin1 treatment, indicating an increase in nuclear Tfeb (Figure 3A,C) [12]. We then evaluated whether the transduction of an adenovirus harboring Tfeb aggravates Tat-Beclin1-induced autotic cell death in NRCMs. As shown previously [9], Tat-Beclin 1 dose-dependently stimulated cardiomyocyte death, most likely by autosis, and the overexpression of Tfeb aggravated Tat-Beclin 1-induced cell death in cardiomyocytes (Figure 3D). In contrast, the knockdown of Tfeb mitigated Tat-Beclin 1-induced cardiomyocyte cell death (Figure 3E). We next investigated whether the Tfeb-induced enhancement of cardiomyocyte cell death is due to autosis. One of the most important features of autosis is that it is prevented by the inhibition of autophagy. The suppression of autophagy via the adenoviral transduction of short hairpin RNA targeting Atg7 (Ad-sh-Atg7) attenuated Tat-Beclin 1-mediated cell death and abolished the augmentation of cell death by Tfeb (Figure 3F–H). Taken together, these results suggest that Tfeb facilitates Tat-Beclin1-induced autotic cell death in NRCMs.

### 3.3. Persistent Activation of Tfeb Triggers Accumulation of Autophagic Vacuoles and Autosis during I/R

**Figure 4:**
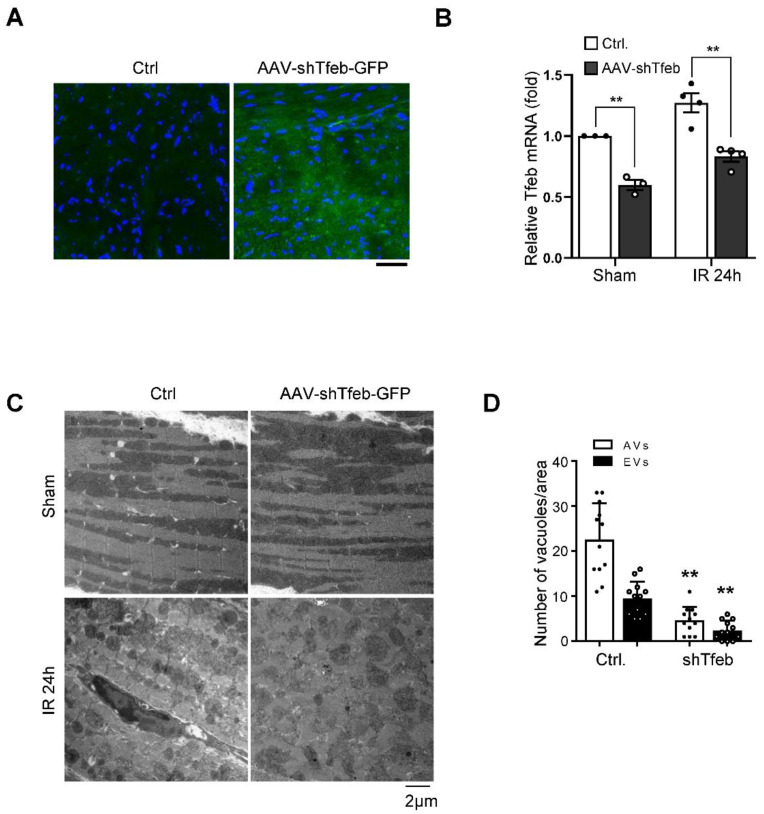
Marked accumulation of autophagic vacuoles is accompanied by upregulation of Tfeb during I/R. (**A**–**D**) AAV9-shTfeb-IRES-GFP was injected into the jugular vein (1.5 × 1012 vg per 10 g for 4 weeks). Four weeks later, mice were subject to 30 min of ischemia and 24 h of reperfusion. The heart samples were subjected to microscopic fluorescent analyses (scale bar = 50 µm) (**A**) mRNA expression of Tfeb in the mouse heart was evaluated (mean values ± S.E., n = 3/each; ** *p* < 0.01) (**B**). (**C**,**D**) The heart samples were subjected to electron microscopic analyses. Autophagic vacuoles (AVs, arrow) and empty vacuoles (EVs) are indicated (scale bar = 2 µm) (**C**). Cytoplasmic AVs and EVs were counted (mean values ± S.D., n = 12; ** *p* < 0.01 versus control with I/R 24 h) (**D**). Experiments were conducted using 3 mice per group.

The persistent activation of Tfeb may induce dysregulated autophagosome accumulation during the late phase of reperfusion since the fusion of autophagosomes to lysosomes is attenuated due to the upregulation of Rubicon [9]. To test this hypothesis, we injected C57BL/6J mice with either AAV9-shTfeb-IRES-GFP or AAV9-shControl, and, after 4 weeks, we applied I/R. Injected AAVs were transduced throughout the heart, as indicated by the GFP signal in frozen sections, and the injection of AAV9-shTfeb-IRES-GFP significantly reduced the expression of Tfeb mRNA compared to AAV9-shControl (Figure 4A,B). EM analyses showed that the total number of autophagic vacuoles was increased during I/R in AAV-shControl-injected hearts. However, I/R-induced increases in the total number of autophagic vacuoles were significantly attenuated in AAV-shTfeb-IRES-GFP-injected hearts (Figure 4C,D).

**Figure 5:**
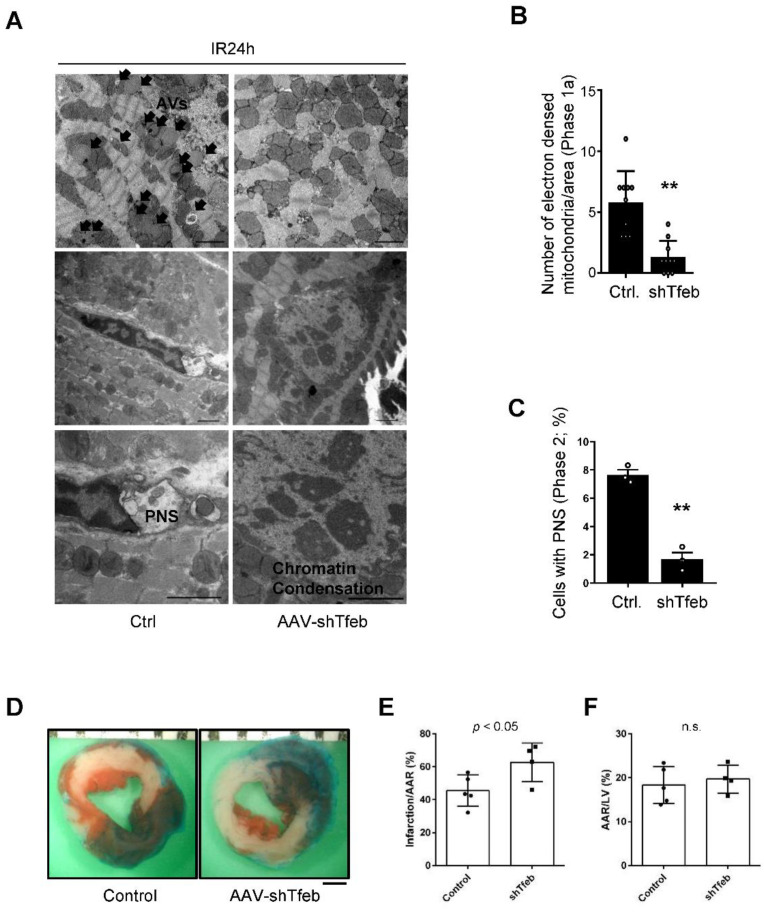
Downregulation of Tfeb attenuates autosis but aggravates I/R injury. (**A**–**F**) AAV9-shTfeb-IRES-GFP was injected into the jugular vein (1.5 × 10^12^ vg per 10 g for 4 weeks). Four weeks later, mice were subject to 30 min of ischemia and 24 h of reperfusion. (**A**–**C**) The heart samples were subjected to electron microscopic analyses. Electron-dense mitochondria and ballooning of the perinuclear space (PNS) are indicated (scale bar = 2 µm) (**A**). The number of electron-dense mitochondria was counted (mean values ± S.D., n = 12; ** *p* < 0.01 versus control with I/R 24 h) (**B**). The percentage of cells with ballooning of the PNS was calculated (mean value ± S.E., n = 3; ** *p* < 0.01 versus control with I/R 24 h) (**C**). Experiments were conducted using 3 mice per group. Representative images of LV myocardial sections after Alcian blue and triphenyl tetrazolium chloride (TTC) staining (scale bar = 1 mm) (**D**). The ratios of the infarction area to area at risk (AAR) (**E**) and AAR to total LV (**F**) are shown (mean value ± S.E., n = 5 (control); n = 4 (shTfeb)).

We next evaluated the level of autosis. EM analyses showed that I/R-induced increases in cardiomyocytes with ballooning perinuclear space (PNS) and electron-dense mitochondria, representative features of autosis, were drastically reduced in AAV9-shTfeb-IRES-GFP-injected mice compared to in AAV-shControl-injected mice (Figure 5A–C). The area at risk (AAR) was not significantly affected by AAV injections (Figure 5D,F). Although injection with AAV9-shTfeb attenuated autosis, the downregulation of Tfeb slightly increased the size of the myocardial infarct compared to injection with AAV9-shControl (Figure 5D,E).

### 3.4. Acute Activation of Tfeb by 3,4-Dimethoxychalcone Aggravates Myocardial Infarction (MI) Caused by I/R

**Figure 6:**
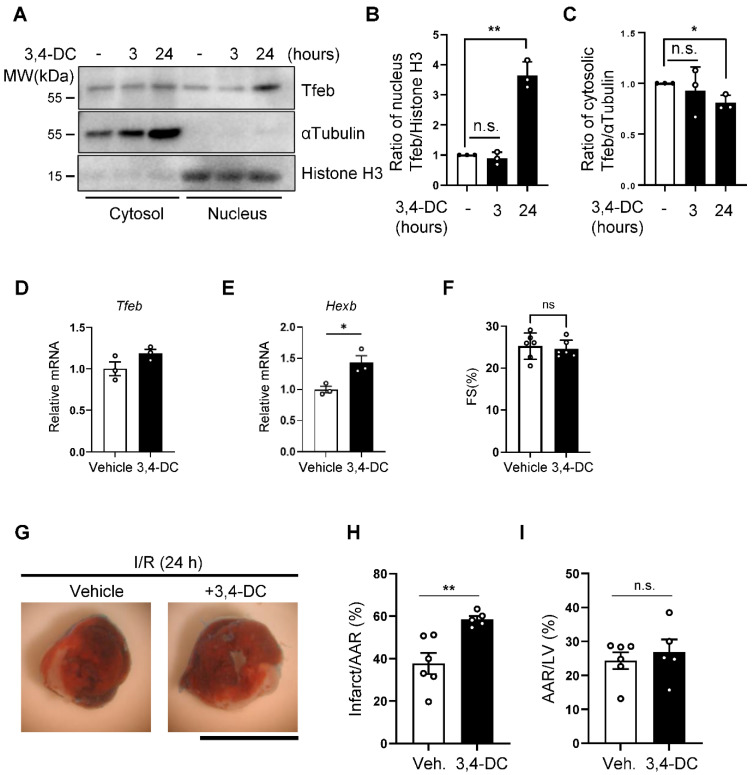
Activation of Tfeb by 3,4-dimethoxychalcone aggravates I/R injury. (**A**–**C**) NRCMs were treated with 30 µM 3,4-dimethoxychalcone (3,4-DC) for indicated time periods and subcellular fractions were analyzed via Western blotting using anti-Tfeb, anti-αTubulin and anti-Histone H3 antibodies (**A**). The ratio of Tfeb to Histone H3 in the nuclear fraction (**B**) and the ratio of Tfeb to αTubulin in the cytosolic fraction (**C**) were quantified (mean values ± S.E., n = 3; ** *p* < 0.01). (**D**,**E**) 3,4-DC was dissolved in corn oil at a concentration of 230 mg/kg and injected intraperitoneally (150 μL/mouse), and 3 h later, whole heart lysates were analyzed via qPCR analysis. Graphs showing relative gene expression levels of Tfeb (**C**) and Hexb (**D**). Mean ± S.E., n = 3; * *p* < 0.05. (**F**) Cardiac function in 3,4-DC- or vehicle-injected mice was evaluated with echocardiographic analyses. Fractional shorting (FS, %), an index of systolic function, is shown (mean ± S.E., n = 6). (**G**–**I**) 3,4-DC was dissolved in corn oil at a concentration of 230 mg/kg and injected intraperitoneally (150 μL/mouse) immediately after reperfusion. Representative images of LV myocardial sections after Alcian blue and triphenyltetrazolium chloride (TTC) staining (scale bar = 1 mm) (**G**). The ratios of the infarction area to area at risk (AAR) (**H**) and AAR to total LV (**I**) are shown (mean value ±S.E., n = 6 (corn oil); n = 5 (3,4-DC); ** *p* < 0.01).

Although the knockdown of Tfeb reduced autosis, the long-term suppression of Tfeb, using AAV-mediated knockdown, exacerbated MI. Since Tfeb-dependent mechanisms, including autophagy, may be salutary during ischemia and the early phase of reperfusion, the persistent downregulation of Tfeb throughout the period of both ischemia and reperfusion could be detrimental even if Tfeb upregulation during the late phase is functionally detrimental. Thus, in order to show the functional significance of Tfeb upregulation during the late phase of reperfusion, it would be ideal to only inhibit Tfeb during the late stage of I/R. However, it is challenging to selectively inhibit Tfeb pharmacologically in a time-restricted manner. We therefore used a gain-of-function approach with 3,4-dimethoxychalcone (3,4-DC), a chemical activator of Tfeb [22]. The treatment of NRCMs with 3,4-DC increased the nuclear level of Tfeb, whereas it decreased the level of Tfeb in cytosol, confirming that 3,4-DC activates Tfeb (Figure 6A–C). qPCR analyses showed that 3,4-DC upregulated a known target of Tfeb, *Hexb,* without affecting Tfeb itself (Figure 6D,E). In order to test whether the enhanced activation of Tfeb during the late reperfusion phase exacerbates I/R injury, we injected 3,4-DC into mice right after reperfusion and evaluated infarction volume. 3,4-DC injection alone did not affect myocardial function (Figure 6F). However, 3,4-DC injection exacerbated myocardial injury (Figure 6G–I), consistent with the notion that the enhancement of Tfeb during the late reperfusion phase promotes I/R injury.

## 4. Discussion

Here, we show that Tfeb is activated during reperfusion after ischemia in the mouse heart. Tfeb activation in the presence of a block of autophagic flux induced the dysregulated accumulation of autophagosome and autosis in cardiomyocytes. On the other hand, the inhibition of endogenous Tfeb attenuated cardiomyocyte autotic cell death during reperfusion in the mouse heart. Thus, the activation of Tfeb contributes to cardiomyocyte autosis during the late phase of I/R.

Small-scale gene expression analyses showed that genes involved in autophagy, including Atg12, Beclin 1, Gabarap and Vps11, and lysosomal function, including Ctsb, Ctsd, Hexb, Cln3 and Atp6v1, are upregulated during myocardial reperfusion in a time-dependent manner. Autophagic flux is stimulated during the early phase of reperfusion but is inhibited during the late phase of reperfusion due to the upregulation of Rubicon [9]. Thus, these genes may participate in stimulating autophagic flux during the early phase of reperfusion but may also serve as a feedback mechanism to stimulate autophagy and lysosomal degradation during the late phase of reperfusion. The transcription of these genes is regulated by Tfeb family transcription factors. In fact, both the mRNA and protein expression of Tfeb were upregulated in response to reperfusion, as was the nuclear expression of Tfeb. Importantly, the accumulation of autophagosomes and autolysosomes during reperfusion was attenuated in the presence of Tfeb knockdown. These results suggest that the activation of Tfeb increases the production of autophagosomes during reperfusion. The continued activation of Tfeb during the late phase of reperfusion, however, induces the uncoupling of autophagosome formation from lysosomal degradation, thereby facilitating the dysregulated accumulation of autophagosomes.

The activity of Tfeb is regulated by both posttranslational and transcriptional mechanisms. Myocardial reperfusion increases the nuclear level of Tfeb. How reperfusion affects the activity of these kinases toward Tfeb remains to be elucidated. We also noted that both mRNA and protein levels of Tfeb are upregulated during reperfusion. The transcription of Tfeb could be regulated not only by PGC-1α and PPARα but also by Tfeb itself [23]. The role of these transcription factors in mediating the upregulation of Tfeb during reperfusion remains to be elucidated. Given that the Tfeb protein level increases during the late phase of reperfusion, it is possible that Tfeb initiates a positive feedback mechanism by which it upregulates itself to cope with the reduced autophagic flux imposed by Rubicon upregulation. Interestingly, our results also show that Tfeb is upregulated by a high dose of Tat-Beclin 1. Thus, a novel mechanism may exist in which Tfeb is upregulated by autosis. If so, together with the induction of autosis by Tfeb (see below), Tfeb and autosis may stimulate one another to form a positive feedback loop.

We have previously shown that the excessive accumulation of autophagosomes during the late phase of I/R induces autosis in cardiomyocytes and that the upregulation of Rubicon inhibits autophagosome–lysosome fusion, thereby mediating the dysregulated accumulation of autophagosomes and autosis in cardiomyocytes. Importantly, the overexpression of Rubicon and the consequent block in autophagic flux alone are not sufficient to induce autosis in cardiomyocytes, suggesting that additional mechanisms, most likely driving the formation of autophagosomes, are required for the induction of autosis. Our results suggest that the upregulation of Tfeb is one such mechanism, promoting autosis in the heart during the late phase of reperfusion.

Here, we show that cardiomyocyte death induced by a high dose of Tat-Beclin 1 [9] was enhanced by Tfeb overexpression but was reversed in the presence of Atg7 knockdown. Thus, the upregulation of Tfeb promotes autosis in cardiomyocytes when autophagy is strongly activated, as during myocardial reperfusion. Furthermore, the high-dose Tat-Beclin 1-induced cardiomyocyte death was attenuated by the downregulation of Tfeb. Together, these results suggest that Tfeb can stimulate autosis in cardiomyocytes when autophagy is strongly stimulated. This appears to also be true in the heart in vivo during myocardial reperfusion. Whereas the downregulation of Tfeb decreased autosis, the stimulation of Tfeb during reperfusion increased the size of MI. Thus, we propose that the upregulation of Tfeb during the late reperfusion phase promotes cardiomyocyte autosis, thereby contributing to reperfusion injury.

Our results suggest that Tfeb could be targeted to prevent the autosis of cardiomyocytes during the late phase of reperfusion. Importantly, however, like the role of autophagy during ischemia and reperfusion, the effect of Tfeb appears to also be time dependent. We have previously shown that the stimulation of autophagy by Tat-Beclin 1 is protective during ischemia and the early phase of reperfusion but is detrimental during the late phase of reperfusion in the heart [9]. Thus, Tfeb should only be targeted during the late phase of reperfusion to prevent autosis but not autophagy during ischemia and the early phase of reperfusion. This could be accomplished by the administration of a chemical inhibitor during the appropriate time period but, unfortunately, a specific chemical inhibitor of Tfeb that can be applied to the heart during the late phase of reperfusion is not currently available. The development of small-molecule inhibitors would expand the options for drug therapy during myocardial reperfusion since autosis has not been targeted previously. The mechanism of autosis appears to be distinct from other forms of cardiomyocyte death during reperfusion. Thus, a therapy targeting autosis may have an additive effect over existing modes of treatment for reperfusion injury.

## Figures and Tables

**Table 1:** Oligo sequences for PCR array of autophagy-related genes.

Gene Symbol	Sense (5′→3′)	Antisense (5′→3′)
Akt1	GCTTCTATGGTGCGGAGATT	CTTGTCCAGCATGAGGTTCT
Ambra1	ATACTACGCCCAGAGGATGA	GAAGAAGAGGAGGTGGAAGAAC
App	TCTCTGTCCCTGCTCTACAA	CAAGACATCGTCGGAGTAGTTC
Atg10	CGAGCGGGTTCTCATTAACA	TCCTTGGCTGTTCTCCATTC
Atg12	AGCTCTTCAGTCCTGTCATTTC	ACTCCTGGTTCACTCTTCCT
Atg16l1	TGTGTGTGTGTGAGGAAAGG	GCAGTGATGCAGGGTAAAGA
Atg16l2	CGAGACAACACACTCAAGGTTA	CAGCTTTGGTCCAGTCAGAA
Atg3	CCGGTCCTCAAGGAATCAAA	GTTGGACAGTGGTGGACTAAG
Atg4a	GAAACGAGGGCATTTGGAAAG	ACCTGAAAGGGTGGAGTAGTA
Atg4b	CAGATGCAGCAGCTCTAAGT	GGGACAAGATATGGGAGAGAAAG
Atg4c	GCGGAGTCTTCTATGAGGAATG	GGAAGGAAGGAAGGAAGGAAAG
Atg4d	CCTGGTCTTGTTGTCTCACTAC	TTCCGCTGGTCCAACTAATC
Atg5	CAGGTGATGATTCACGGGATAG	GGAGGACACACTCTTTCAATCT
Atg7	TCCTGAGAGCATCCCTCTAAT	GGCTCGACACAGATCATCATAG
Atg9a	GACATCTACCATCGCATCCTAC	GGGTGAAGAAGACAACCTCTC
Atg9b	GATGACAGGCCCAGGATATTT	GCCAGGCTAGTGTTCTCTTATC
Bcl2	GAGCAGGTGCCTACAAGAAA	CTTTGTCCTCTGACTGGGTATG
Bcl2l1	CTGGTTGAGCCCATCTCTATT	CTGACTCCAGCTGTATCCTTTC
Becn1	CAGGAACTCACAGCTCCATTAC	CCATCCTGGCGAGTTTCAATA
Bnip3	TCCAGCCTCCGTCTCTATTT	CTGTCACAGTGAGAACTCTTGG
Cdkn1b	CCTTCCGCCTGCAGAAAT	CTGACTCGCTTCTTCCATATCC
Cdkn2a	CATGTTGTTGAGGCTAGAGAGG	CACCGTAGTTGAGCAGAAGAG
Cln3	CCACCTGTCCCAGACTTTAATC	CCGCAGAACTTCCCATAACA
Ctsb	CATGCACTGGAGAAGGAGATAC	CTGTTAGACACGCTGTAGGAAG
Ctsd	CCTGGGCGATGTCTTCATT	GTGGAGAAGGAGCAAGTTAGAG
Ctss	GGGAGACATGACCAATGAAGAA	TGTCAGGCAATGTCCGATTAG
Cxcr4	CTGCCCACCATCTACTTCATC	CGTCATGCTCCTTAGCTTCTT
Dapk1	CAACAGGGCTTCTAGGGATTT	AGCTCCCGAAGTTTGGATATAAG
Dram1	CAGATCCAAGTCAGGGAGAAAG	CAGGCATTGATAGGAGCAGATAA
Dram2	TGGTAGTGCACGCCTTTAAT	CTGTCCTGGAACTCATTCTGTAG
Eif2ak3	CCCAGGCATTGTGAGGTATT	CCAGTCTGTGCTTTCGTCTT
Eif4g1	GCTACAAGCACTCTATGCTCTC	CTTCACCACGTCCTCATCATATAG
Esr1	CTTGGAAGGCCGAAATGAAATG	GGCAGGGCTATTCTTCTTAGTG
Gaa	CCAATTCCTCTCCACACACTAC	TTGAGCGGGAGATCACAAAG
Gabarap	CTCCTTCCTTGACATCCAGTTC	GGAGAGACAGCCTCAAACATTA
Gabarapl1	CCTAACCTCTGTCTCCATACCT	GAATGTCTCCTGCCACAACT
Gabarapl2	GCACTGTCCTCATGGCTATT	CACACCTACCTCCCTTCATTAC
Hdac1	GCTGCTCAACTATGGTCTCTAC	CACTGTGGTACTTGGTCATCTC
Hdac6	GACCTGTCTACCTGGTGTTATG	GCAGTGTGGTCTGGGATTTA
Hgs	TGAGGAGAAAGAGAGGATGAGA	GTTCACAGGCGAGGAATACA
Hsp90aa1	GACGAGATGGTTTCTCTGAAGG	TTCCACAAAGGCGGAGTTAG
Hspa8	ACTCCTCTTTCCCTTGGTATTG	GTCAGAGTAGGTGGTGAAAGTC
Ifng	CTCTTCCTCATGGCTGTTTCT	TTCTTCCACATCTATGCCACTT
Igf1	CAGAAGAGGGAGAGAGAGAGAA	TAGCAAGCAGAAGAGGGATTTAG
Ins2	CCCTAAGTGATCCGCTACAATC	CAGGGCCATGTTGAAACAATAA
Irgm1	CGAAGACCAGAAGCTGAAAGA	CCGGATGTTGAGAGATGAGATG
Lamp1	GACCCTGAAAGTGGAGAACAA	GGGCATCAGGAAGAGTCATATT
Map1lc3a	GTCTACGCCTCCCAAGAAAC	GGTGTCACATCTCTGCCTAATC
Map1lc3b	CGCATGCCTTTAGCCTTTAATC	CCTGGAACTCACTTTGTAGACC
Mapk14	GAAAGCAGGGACCTTCTCATAG	GTGCTCAGGACTCCATTTCTT
Mapk8	TCCAGCACCCATACATCAAC	CTATTGTGTGCTCCCTCTCATC
Mtor	CGGGACTACAGAGAGAAGAAGA	CATCAACGTCAGGTGGTCATAG
Nfkb1	GGGATTTCGATTCCGCTATGT	GACCTGTGGGTAGGATTTCTTG
Npc1	GAGCAGATCCTGTCTGCATTAT	CTGGTGTATCCGTGAAGTGTT
Pik3c3	CCTGATGCCTGGCTCAATAA	TAACTTCTGGACACCCTCTCT
Pik3cg	GTACCCTGGTGATCGAGAAATG	ATGGTTTCGTTGGATAGGACTG
Pik3r4	AGGTTCTGGGACTTGGTTTC	GACCTCGGTGCCTTCTATTATC
Prkaa1	TGGCTGGGTGTGTAAAGATATG	CATGTCAGAGCCCACAATGA
Pten	CAGTGAATGCCATCACCATTTC	CTGGGCTTAAGGTCTGATTCTC
Rab24	ATCTGGCCCAGTGGAATTAG	GTGAGAGGCACCTGGAATAG
Rb1	CCTCCTACCTTGTCACCAATAC	GTTACCTCCAGGAATCCGTAAG
Rgs19	TGCGAGAAGGCATCAATAGG	GGAGTAATAGAGAGCGGTAGGT
Rps6kb1	AGAGATGGGTCAGGGTTAAGA	TTCAGGTTCCCAGGAAGTAAAG
Snca	TGACAACAGTGGCTGAGAAG	CCAGTGGCAGCAGCTATATT
Sqstm1	CTCTGGACACGATCCAGTATTC	CTGCTCTACGTGATGCAACTA
Tgfb1	GGTGGTATACTGAGACACCTTG	CCCAAGGAAAGGTAGGTGATAG
Tgm2	CATCACCAGCACTCTGTATCTC	GGTTCCTTCGGTTCCTTCAT
Tmem74	TGCTCCAAGACAGTGCTATTC	GTTCCTGGCTGCAACAATTAC
Tnf	TTGTCTACTCCCAGGTTCTCT	GAGGTTGACTTTCTCCTGGTATG
Tnfsf10	CAGCCCTAAAGTACCCAGTAATC	CACATCTGTCCTGAGGTTTCTAC
Trp53	CAGTCTACTTCCCGCCATAAA	GTCTCAGCCCTGAAGTCATAAG
Ulk1	CAGGGTGGACACATGCTAATAC	CAGCTTGTGGACACTCAGATAC
Ulk2	CAACATCTCGTCAGACCACTC	CACTGCCCTCCACACATAAA
Uvrag	GACCACGAGACAGTTGAGATAG	GCAGGGACAATGGACTTAGAA
Wipi1	GTGTGTCTAGACGACGAGAATG	GACTTCTGAGGTAGGCTTCTTG
